# Supplementary material for: PTEN and DNA-PK determine sensitivity and recovery in response to WEE1 inhibition in human breast cancer
Source: eLife. 2020 Jul 6;9:e57894. doi: 10.7554/eLife.57894 (PMC7338058; doi:10.7554/eLife.57894)
Supplement: Transparent reporting form [file elife-57894-transrepform.docx]

***eLife’s* transparent reporting form**

We encourage authors to provide detailed information *within their submission* to facilitate the interpretation and replication of experiments. Authors can upload supporting documentation to indicate the use of appropriate reporting guidelines for health-related research (see [EQUATOR Network](http://www.equator-network.org/%20)), life science research (see the [BioSharing Information Resource](https://biosharing.org/)), or the [ARRIVE guidelines](http://www.plosbiology.org/article/info:doi/10.1371/journal.pbio.1000412) for reporting work involving animal research. Where applicable, authors should refer to any relevant reporting standards documents in this form.

If you have any questions, please consult our Journal Policies and/or contact us: [editorial@elifesciences.org](mailto:editorial@elifesciences.org).

**Sample-size estimation**

- You should state whether an appropriate sample size was computed when the study was being designed
- You should state the statistical method of sample size computation and any required assumptions
- If no explicit power analysis was used, you should describe how you decided what sample (replicate) size (number) to use

Please outline where this information can be found within the submission (e.g., sections or figure legends), or explain why this information doesn’t apply to your submission:

The design of the study was exploratory, inferring novel findings from data that was not available elsewhere. No statistical sample size determination or power analysis was therefore utilized. The criteria used for sample selection (human breast cancer-derived cell lines) was based on transcriptionally defined molecular breast cancer subtypes (i.e. luminal and basal-like BCCLs). The mouse experiments were conducted according the guidelines of the Karolinska Institute animal facilities and according to the 3R rules. The number of mice per group were therefore kept to a minimum. The methods, size of groups and statistical analysis applied are explained in the materials and methods section as well as in the figure legends.

**Replicates**

- You should report how often each experiment was performed
- You should include a definition of biological versus technical replication
- The data obtained should be provided and sufficient information should be provided to indicate the number of independent biological and/or technical replicates
- If you encountered any outliers, you should describe how these were handled
- Criteria for exclusion/inclusion of data should be clearly stated
- High-throughput sequence data should be uploaded before submission, with a private link for reviewers provided (these are available from both GEO and ArrayExpress)

Please outline where this information can be found within the submission (e.g., sections or figure legends), or explain why this information doesn’t apply to your submission:

Technical replicates were considered identical samples (analysed in parallel), while biological replicates were considered to be experiments performed on distinct samples as reported in figure legends where applicable. Drug response (i.e. proliferation, viability, DNA damage), proteomics and transcriptomics were performed in triplicate technical replicates. The raw data and processed data used for analysis are available for download at: MS proteomics data is uploaded to ProteomeXchange with the identifier PXD013276, RNA-seq data to NCBI GEO with the identifier GSE152102. Additionally, processed data used for analysis is also provided as supplementary files (Suppl file_2_protein, Suppl_file 3_mRNA).

High-content image (HCI) drug and siRNA screening was performed as stated in the figure legends and methods sections (Processed data shown in Figure 1 and 4).

The mouse experiments are explained in the materials and methods section as well as in the figure legends.

In general, all biochemical and molecular experiments were performed at least three times. Molecular data (immunoblots, FACS, immunofluorescence) generated using isogenic knockout cell lines (clones) is based on at least two independent biological replicates. Outliers were included in the figures and described in the text.

**Statistical reporting**

- Statistical analysis methods should be described and justified
- Raw data should be presented in figures whenever informative to do so (typically when N per group is less than 10)
- For each experiment, you should identify the statistical tests used, exact values of N, definitions of center, methods of multiple test correction, and dispersion and precision measures (e.g., mean, median, SD, SEM, confidence intervals; and, for the major substantive results, a measure of effect size (e.g., Pearson's r, Cohen's d)
- Report exact p-values wherever possible alongside the summary statistics and 95% confidence intervals. These should be reported for all key questions and not only when the p-value is less than 0.05.

Please outline where this information can be found within the submission (e.g., sections or figure legends), or explain why this information doesn’t apply to your submission:

Exact p-values were reported in text, figures and legends.

Statistical analysis was performed GraphPad Software as described in the method section. Statistical tests being used are described in the corresponding figure legends and method section.

Multiple test correction was performed where deemed appropriate. For comparison of 2 groups Student’s t-test was used. For comparision of multiple protein groups, one-way Anova was used with correction for multiple testing according to Tukey.

(For large datasets, or papers with a very large number of statistical tests, you may upload a single table file with tests, Ns, etc., with reference to sections in the manuscript.)

**Group allocation**

- Indicate how samples were allocated into experimental groups (in the case of clinical studies, please specify allocation to treatment method); if randomization was used, please also state if restricted randomization was applied
- Indicate if masking was used during group allocation, data collection and/or data analysis

Please outline where this information can be found within the submission (e.g., sections or figure legends), or explain why this information doesn’t apply to your submission:

In the result section (figure 1), you will find the information on the different response groups of breast cancer cell lines.

In the mouse experiments, 4-6 mice were allocated in each group in order to keep the number of mice as low as possible according to the 3R rules at the Karolinska Institutet. The statistical analysis is explained in the figure legends.

**Additional data files (“source data”)**

- We encourage you to upload relevant additional data files, such as numerical data that are represented as a graph in a figure, or as a summary table
- Where provided, these should be in the most useful format, and they can be uploaded as “Source data” files linked to a main figure or table
- Include model definition files including the full list of parameters used
- Include code used for data analysis (e.g., R, MatLab)
- Avoid stating that data files are “available upon request”

Please indicate the figures or tables for which source data files have been provided:

HCI drug response raw data is provided as source data file 1 and processed data shown in Figure 1. HCI RNAi data have been deposited at Mendeley (https://data.mendeley.com/), DOI 10.17632/rmjnmwzmf6.1.

Processed proteomic and transcriptomic data are provided as supplementary files (see above). All data is preserved for transparency as source data files and have been uploaded to GEO GSE152102 and ProteomeXchange PXD013276.

All numerical data can be derived from the figure supplements, deposited data files, or from source data files linked to the main figures.
